# 1088. Evaluating the Effect of Donepezil on Mortality Among Alzheimer’s Disease Patients With and Without COVID-19 Infection

**DOI:** 10.1093/ofid/ofac492.928

**Published:** 2022-12-15

**Authors:** Taissa A Bej, Elizabeth Edmiston, Brigid Wilson, Joy Phillips, Robin L Jump

**Affiliations:** Louis Stokes Cleveland VA Medical Center, Cleveland, Ohio; VA Northeast Ohio Healthcare System, Cleveland, Ohio; Louis Stokes Cleveland VA Medical Center, Cleveland, Ohio; San Diego State University, San Diego, California; University of Pittsburgh School of Medicine, Cleveland, Ohio

## Abstract

**Background:**

Dementia has been identified as an independent risk factor for increased severity of COVID-19 infection. Donepezil, a cholinesterase inhibitor approved for Alzheimer’s disease (AD), has anti-inflammatory properties. Previous studies have found that donepezil reduced all-cause mortality for people living with AD. The anti-inflammatory effects of donepezil have not been studied in patients with COVID-19 and AD. Here, we compare mortality rates of patients with AD to assess the impact of donepezil on the severity of COVID-19 infections.

Survival following SARS-CoV2-2 test, stratified by result and Donepezil

**Figure:**
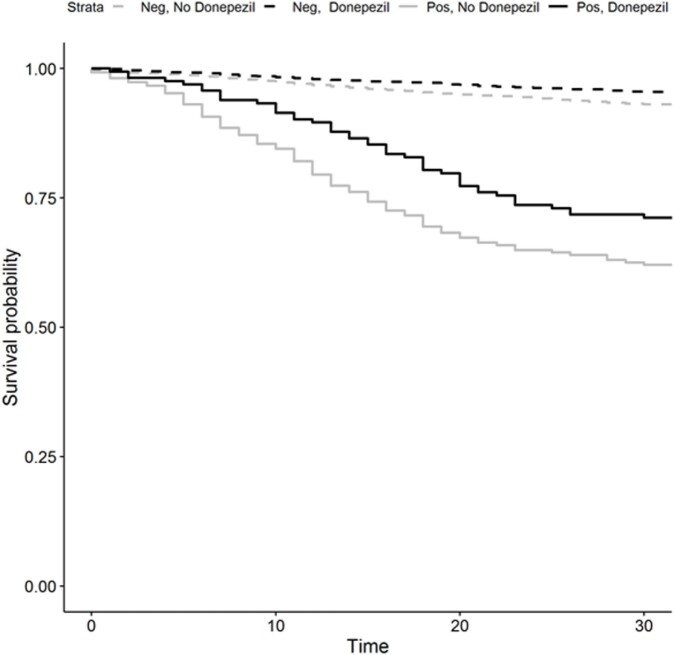

Kaplan-Meier curves of all-cause mortality for Veterans with Alzheimer’s Disease taking donepezil (black) compared to those who were not taking donepezil (grey), further stratified by those with a positive SARS-CoV-2 PCR test (solid lines) or no evidence of a COVID-19 infection during the study period (dashed lines). While donepezil has a positive effect on survival, the adjusted survival odds ratio is not greater among those with vs. without a COVID-19 infection.

**Methods:**

Using administrative data from the Veterans Healthcare Administration (VHA), we conducted a national retrospective cohort study of Veterans with AD who were tested for SARS-CoV-2 between March 1, 2020 and December 31, 2021 in the VHA. Among these patients, we assessed all-cause 30-day mortality stratified by COVID-19 infection and donepezil use and considered the interaction of these two factors. For Veterans with a positive test, the date of first positive test was used to assess mortality; for Veterans without a COVID-19 diagnosis or positive test, date of first negative test was used.

**Results:**

During the study period, 582 Veterans with Alzheimer’s disease were positive for COVID-19 and 14430 had no test or diagnosis indicating COVID-19 infection. Among people with AD and COVID-19, all-cause 30-day mortality was 29% (47/163) for people taking donepezil compared to 38% (159/419) for those who were not. Among people with AD without COVID-19, all-cause 30-day mortality was 5% (189/4189) for people taking donepezil compared to 7% (712/10241) for those who were not. In a multivariable logistic regression, the decrease in mortality associated with donepezil did not differ between people with and without COVID-19 (OR (95% CI) = 0.71 (0.47, 1.07) vs. OR (95% CI) = 0.68 (0.57, 0.80), interaction P = 0.818).

**Conclusion:**

While all-cause mortality was lower for patients taking donepezil compared to those not taking donepezil, the protective effect of donepezil was not increased in AD patients with COVID-19 over those without COVID-19. The population differences and inflammatory biomarkers of AD patients treated with and without donepezil merit further study.

**Disclosures:**

**Robin L. Jump, MD, PhD**, Merck: Grant/Research Support|Pfizer: Advisor/Consultant.

